# T91. DEVELOPMENT OF NOVEL BIS-AMIDINES FOR THE TREATMENT OF TOXOPLASMOSIS

**DOI:** 10.1093/schbul/sby016.367

**Published:** 2018-04-01

**Authors:** Lorraine Jones-Brando, Claudia Bordón, Robert H Yolken, Varvara Misheneva, Mikhail Pletnikov, Omkar Revu, James McNulty

**Affiliations:** 1 Johns Hopkins University School of Medicine; 2 McMaster University

## Abstract

**Background:**

Toxoplasma infections constitute a worldwide public health problem responsible for significant morbidity. Symptoms of acute infection by the agent, the apicomplexan parasite Toxoplasma gondii, can range from mild to life-threatening depending upon the immune status of the host. Toxoplasma infections are often unrecognized in immune competent hosts but infection can lead to the long-term establishment of cysts in the brain and thus a persistent infection. Such tissue cysts are associated with altered behavior and cognition in mouse models. Furthermore, serological evidence of Toxoplasma infection has been associated with an increased risk of recent onset psychosis, schizophrenia, and other psychiatric disorders in humans. Adjunct treatment of Toxoplasma-seropositive individuals afflicted with schizophrenia with effective anti-parasitic medications could be beneficial by eliminating the parasite cysts, thereby possibly alleviating psychiatric symptoms. However, currently available medications are ineffective against the cyst form of the organism responsible for persistent infection. Others have published the anti-Toxoplasma potential of derivatives of the bis-amidine pentamidine. We designed and synthesized a panel of novel bis-amidines and explored their anti-Toxoplasma efficacy.

**Methods:**

We designed and synthesized an array of twelve novel bis-amidines. These compounds were examined for in vitro activity against T. gondii tachyzoites first using a colorimetric assay employing the β-gal producing strain RH-2F to examine the effect of the compounds over 5 days of parasite growth in human fibroblast host cells. This was followed by several immunofluorescence-based assays to determine if compounds can directly act on tachyzoites (red/green invasion assay), on an established infection of host cells (replication assay), and whether the compounds are parasiticidal, ie. can rid the host cells of tachyzoite infection after one dose (recovery assay). A rodent model of acute toxoplasmosis was established for examining in vivo efficacy of lead compounds.

**Results:**

Four of these compounds proved highly efficacious in vitro with 50% inhibitory (IC50) values ranging from 200 nM to 1.3 µM. Therapeutic indices based on the ratio between the median cell cytotoxic dose and the IC50 ranged from 4 to 84. These four compounds all inhibited tachyzoite invasion and significantly inhibited in vitro replication over a 24-hour period at nanomolar concentrations. Additionally, two of the compounds evaluated thus far appear to be parasiticidal in vitro at 1 - 2 µM.

**Discussion:**

Our results suggest that bis-amidines can be designed to be effective against experimental Toxoplasma infections. The parasiticidal activity of some of the compounds make them serious candidates for further drug development. Further experiments to determine 1) in vivo efficacy in mouse models of persistent infection, and 2) synergy with antipsychotics and mood stabilizers will investigate the possibility that these compounds could be used as adjunct treatment in Toxoplasma-positive individuals suffering with schizophrenia and other psychiatric disorders.

